# Acute interstitial nephritis observed with three different triggering agents

**DOI:** 10.1002/ccr3.5432

**Published:** 2022-03-18

**Authors:** Niloofar Nobakht, Ramy M. Hanna, Mohammad Kamgar, John Sinclair, Lewis Simon, Sina Emami, Anthony Sisk, Anjay Rastogi

**Affiliations:** ^1^ Department of Medicine Division of Nephrology David Geffen School of Medicine (DGSOM) Los Angeles California USA; ^2^ School of Medicine University of California Irvine Orange California USA; ^3^ 3239 Lake Erie College of Osteopathic Medicine Erie Pennsylvania USA

**Keywords:** AIN, AKI, ATN, bromhexine, NSAIDS, PPI

## Abstract

A 70‐year‐old female patient developed acute interstitial nephritis (AIN) after treatment with non‐steroidal anti‐inflammatory drugs (NSAIDs), proton pump inhibitors (PPI), and Bromhexine. Renal biopsy confirmed the diagnosis, and the patient was treated with oral prednisone. Careful attention to timing of acute kidney injury (AKI) is crucial to diagnosing AIN.

## Graphical abstract

Several drugs can cause allergic interstitial nephritis (AIN) including over the counter agents. Bromhexine an over the counter cough remedies can be associated with AIN. The timing of treatment initiation with steroid is critical to decrease risk of fibrosis and future chronic kidney disease and loss of GFR.

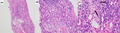

## 1 INTRODUCTION

AIN is challenging to diagnose as it rarely presents with the classically recognized triad of rash, fever, and eosinophilia.^1^ This classic presentation only occurs in less than 5%–10% of patients. Eosinophiluria, though classically described, is an insensitive and nonspecific sign of AIN.^2^, ^3^, ^4^ The best diagnostic technique for AIN is a kidney biopsy,^5^, ^6^, ^7^, ^8^ and this can sometimes be relatively contraindicated in patients with bleeding diathesis or need for anticoagulation therapy. The individual components of the triad also occur in relatively low percentages.^6^

50% of patients with AIN develop a fever, <10% develop a rash, and only 33% develop eosinophilia. This should lead to a high index of suspicion should a patient experience any of these symptoms individually along with renal dysfunction.^5^

Proton pump inhibitors (PPI) are widely used and are available in certain doses without a prescription to treat gastrointestinal disorders and discomfort. While they are readily available over the counter (OTC), they are still able to produce adverse effects. PPIs have been associated with adverse reactions like hyponatremia, hypomagnesemia, calcineurin inhibitor‐related drug interactions, and AIN, with hyponatremia and AIN being among these side effects.^1^, ^9^

Nonsteroidal anti‐inflammatory drugs (NSAIDs) are another OTC class of widely used drugs that are notorious inducers of renal injury in general and acute interstitial nephritis in certain cases.^10^ NSAID‐induced AIN is thought to occur through the inhibition of the enzymes cyclooxygenase‐1 (COX‐1) and COX‐2. These enzymes in conjunction serve to synthesize prostaglandins from arachidonic acids.^10^ The products of COX‐1 are constitutively synthesized and serve protective and homeostatic functions. The inhibition of COX‐2 is what gives NSAIDs their analgesic properties, but NSAIDs do not selectively inhibit COX‐2 over COX‐1 to varying degrees.^10^ This is why NSAIDs can be so nephrotoxic, as COX‐1 products are needed during times or incidences of reduced renal perfusion.^10^

Hepatic cirrhosis and patients with cardiac dysfunction are at even greater risk for compromised renal function, and as such, NSAIDs are doubly dangerous in these patient populations.^11^ In hypo‐perfused kidneys, COX‐1 restores perfusion by dilating vascular beds to enhance organ perfusion.^11^

There are many other agents capable of causing AIN; in this case, we discuss a patient who developed brisk AKI while using PPI, NSAIDs, and Bromhexine‐based over the counter cough medication. Bromhexine is a long used mucolytic that has been used in these formulations for relief from sinus and chest congestion. The first two agents are well reported, but Bromhexine has been reported to cause AIN only in sporadic case reports.^12^, ^13^ The challenge of diagnosing AIN was compounded by having three suspected triggering agents, all of which had been previously reported to have caused AIN in various patients in the literature. Furthermore, the patient presented with fever, which was thought to be attributed to possible infectious etiologies at first glance.

## 2 CASE REPORT

We present the case of a 70‐year‐old female patient with a past medical history of dyslipidemia, hypertension, persistent asthma, glucose intolerance, and obesity (Body Mass Index of 32.37 kg/m^2^ at time of presentation). Three weeks prior to presentation, the patient contracted severe food poisoning while on vacation, which the patient attributed to a single, particular meal that the patient had consumed the day prior.

The patient had consistently taken OTC medications in the time leading up to presentation, including: Esomeprazole for 3 months, Bromhexine Hydrochloride for four months, Cetirizine for eight months, and the NSAID Naproxen for 48 h. As part of her hypertension and dyslipidemia treatment, the patient had also been taking prescribed Benazepril and Atorvastatin prior to presentation.

The patient was referred to Nephrology for AKI with a serum creatinine level of 2.1 mg/dl at time of presentation, significantly elevated up from 0.87 mg/dl two months prior (Normal range 0.7–1 mg/dl). This increase in serum creatinine was paired with a corresponding decrease in estimated glomerular filtration rate (eGFR), from 85 ml/min one month prior to presentation to 27 ml/min at the time of presentation (Normal range 90–120 ml/min).

Given her past history of food poisoning and volume depletion, the patient was given intravenous (IV) saline prior her nephrology visit, which only minimally helped with GFR improvement. Following this IV saline‐driven drop in serum creatinine, the patient was discharged from the ED. However, when the patient was seen four days later for a standard follow‐up appointment, her serum creatinine had increased back up to 2.23 mg/dl, higher than her original serum creatinine level of 2.1 mg/dl seen at her previous presentation to the Emergency Department.

Notable laboratory data findings included C‐reactive protein (CRP) level of 2.8 mg/dl (normal range <10 mg/L), erythrocyte sedimentation rate (ESR) of 49 mm/h (normal range 0–20 mm/h), blood urea nitrogen (BUN) level of 25 mg/dl, creatinine of 2.23 mg/dl at presentation and 1.97 mg/dl 5 days later after again being given IV saline, eGFR of 25 ml/min, proteinuria (1+; normal not present), urinalysis leukocyte esterase (2+; normal not present), urinalysis white blood cells (WBC) of 138 cells/µl and 13 cells/high power field (HPF; normal range 0–5 cells/hpf), and urinalysis eosinophils positive at >6% (normal not present). Urine WBC 67 cells/µl with eosinophils >6% were highly suggestive of acute interstitial nephritis (AIN).

Once an active urinary tract infection (UTI) was ruled out with negative urine culture, the patient underwent a kidney biopsy which revealed 11 glomeruli, of which 2 were obsolescent. 30–40% of the renal cortex showed scarring and interstitial fibrosis and tubular atrophy (IFTA). There were no findings of crescents, necrosis, or other deposits noted. A marked mononuclear infiltrate was seen, and these are identified as lymphocytes, plasma cells, and eosinophils. Tubulitis was noted, and there were no signs of arteriolar injury. Immunofluorescence was negative to C1q, C3, IgM, IgG, and IgA.

**FIGURE 1:**
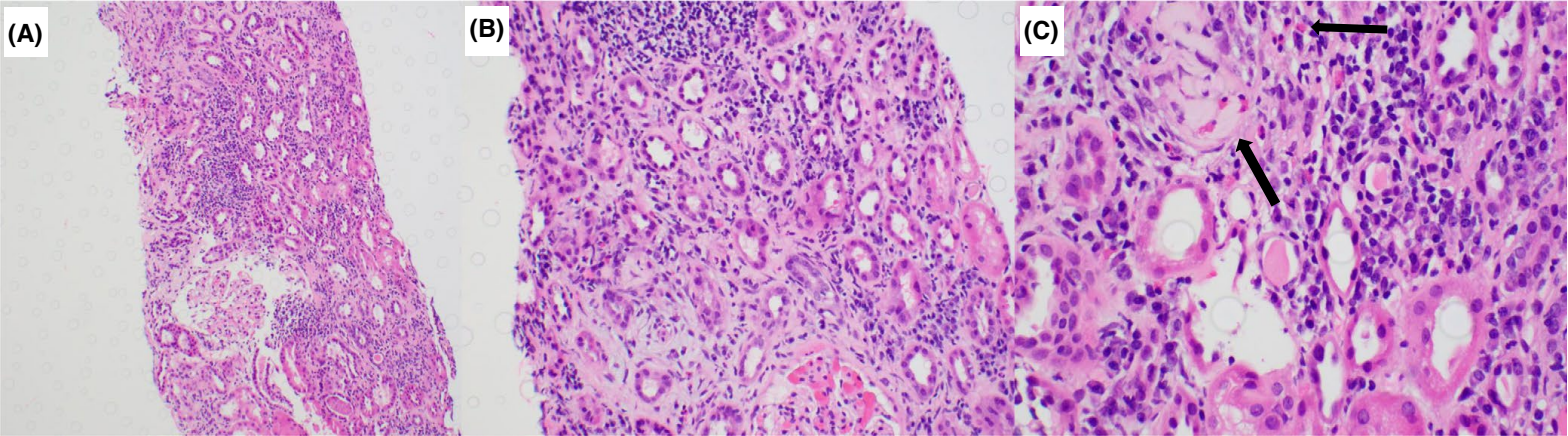
Renal biopsy showing AIN. Legend: (A) 40× magnification. Hematoxylin and eosin (H&E) stain. Inflamed cortex. (B) 200× magnification. H&E stain. Inflammation within scarred cortex. (C) 400× magnification. H&E stain. Mixed inflammation involving non‐scarred cortex. Note eosinophils next to a ruptured tubule (red arrows)

Electron microscopy ultrastructural evaluation was done on two glomeruli, confirming tubular scarring and inflammation. Podocytes were noted to have intact foot processes. Basement membranes were mildly wrinkled but normal contours and endothelial cells were unremarkable. There were no tubuloreticular structures, immune complex deposits, or mesangial abnormalities in any location. A final diagnosis of chronic active tubulointerstitial nephritis was noted with moderate tubular atrophy and interstitial fibrosis. Incidental note was made of mild arterial nephrosclerosis. Please see Figure 1 for renal biopsy findings. Prednisone was started given the patient's acute kidney injury and the clinical clarity of the AIN diagnosis.

**FIGURE 2:**
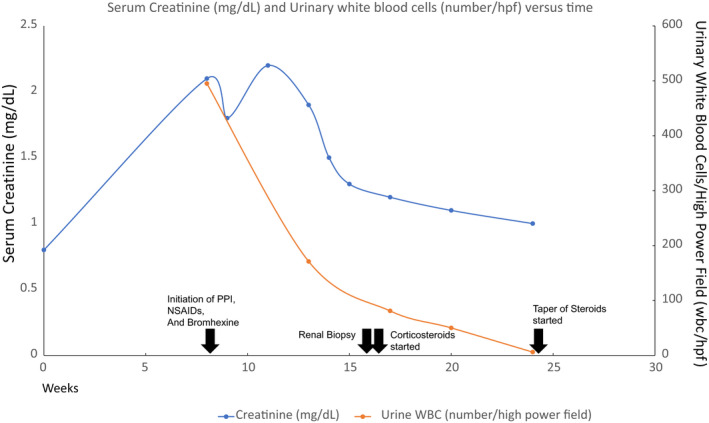
Serum creatinine (mg/dl) and urinary white blood cells/high power field (hpf) versus time. Arrows with description of event added

The patient started on oral steroid treatment in a dose of 40 mg with prophylactic Trimethoprim/sulfamethoxazole (TMP‐SMX) for pneumocystitis jiroveci pneumonia (PJP, formerly PCP) prophylaxis and famotidine which was tapered 6–12 weeks. It is important to note that while starting the patient on TMP‐SMX for PJP prophylaxis is common practice in cases such as these, there is a small inherent risk that TMP‐SMX can cause simple elevation in creatinine levels.^17^ Fortunately, in this case, after initiating these treatments, the patient's renal function started to improve, with serum creatinine levels decreasing from 2.2 mg/dl at presentation to 1.9 to 1.5 to 1.3 to 1.2 within the span of 32 days. See Figure 2 for details of blood and urinary test result trends. Urinary white blood cell findings also started to improve (urine WBC 138–67–2). Though not a very sensitive measure, urinary eosinophils also normalized from >6% to low positive <5% to negative in 6 months. Prednisone was tapered over 8 weeks, and 6 months later, the patient's serum creatinine decreased to 0.8 mg/dl after 6 months.

## 3 DISCUSSION

Despite a relatively low rate of AIN in patients on NSAIDS, the sheer scope of their use means that the 1%–5% of patients taking NSAIDs daily developing renal injury translates into 500,000–2.5 million patients with NSAID‐induced AKI per year.^14^

Instead of the usual triad of AIN in NSAID‐induced interstitial nephritis, the patient experienced edema, proteinuria, and foamy urine, while also reporting decreased urine output initially which improved with IVF. The urine microscopy is often modestly active and contains microscopic hematuria as well as tubular epithelial cell casts. Kidney biopsy is important in the diagnosis of NSAID‐induced AIN,^5^ and sometimes can reveal other glomerular disorders like minimal change disease, membranous glomerulonephritis, and rarely membranoproliferative glomerulonephritis.^15^

In most cases of NSAID‐induced AIN, simply removing the antagonizing NSAID can lead to improvements in kidney function.^16^ Multiple studies have shown that steroid treatment has significant benefits in patients with AIN, with a greater improvement in eGFR and lower risk of progressing to ESRD compared to patients who did not receive steroid treatment.^18^ Gonzalez et al. recommend that these steroids should be started promptly after diagnosis of AIN to avoid subsequent interstitial fibrosis, in addition to an incomplete recovery of renal function.^19^ This case was on a combination of NSAIDs, PPI, and Bromhexine which caused the AKI due to AIN. We believe prompt the initiation of steroid treatment was associated with the recovery of renal function to normal baseline as the response of GFR improvement paralleled treatment timeline over 12 weeks with a great outcome to baseline creatinine which can prevent the transition to chronic interstitial fibrosis and potentially permanent loss of renal function.

This report highlights a complex case with various OTC Medication triggering of what clinically was apparent as drug‐induced AIN with an acute GFR loss with AKI. Early recognition of AIN is an important matter in treating over the counter medication associated AIN to prevent subsequent fibrosis and scaring which may cause permanent renal function loss. This case also highlights the importance of close monitoring of patients routinely taking PPIs along with other medications such bromohexine and NSAIDS, and agrees with Xie et al. (2016) in limiting the overall use of PPI due to AIN and CIN risk.^9^

## CONFLICT OF INTEREST

None.

## AUTHOR CONTRIBUTIONS

NN was first author and lead writing of manuscript. RMH cowrote manuscript. MK contributed to introduction. JS and LS contributed to case report. SE contributed to figures. AS contributed to pathology figure. AR is senior author and edited/reviewed manuscript.

## ETHICAL APROVAL

This research work does not contain human subject research material, as it is an individual anonymized case report. IRB permission was not applied for as it is not required for individual case reports.

## CONSENT

The consent was obtained from the patient to publish this report in accordance with the journal's patient consent policy.

## Data Availability

Not applicable, no data.
